# Immune-related adverse events and atypical radiological response with checkpoint inhibitor immunotherapy in an elderly patient with high PD-L1 expressing lung adenocarcinoma

**DOI:** 10.18632/oncotarget.25984

**Published:** 2018-08-31

**Authors:** Eduard Teixidor, Elia Sais, Carmen Amalia Vásquez, Walter Carbajal, Alejandro Hernández, Gloria Sánchez, Angel Izquierdo, Sara Verdura, Javier A. Menéndez, Joaquim Bosch-Barrera

**Affiliations:** ^1^ Department of Medical Oncology, Catalan Institute of Oncology, Doctor Josep Trueta University Hospital, Girona, Spain; ^2^ Department of Anatomical Pathology, Dr. Josep Trueta Hospital of Girona, Girona, Catalonia, Spain; ^3^ Department of Radiology, Diagnostic Imaging Institute, Doctor Josep Trueta University Hospital, Girona, Spain; ^4^ ProCURE (Program Against Cancer Therapeutic Resistance), Metabolism and Cancer Group, Catalan Institute of Oncology, Girona, Catalonia, Spain; ^5^ Girona Biomedical Research Institute (IDIBGi), Girona, Catalonia, Spain; ^6^ Department of Medical Sciences, Medical School, University of Girona, Girona, Spain

**Keywords:** immunosenescence, elderly, nivolumab, lung cancer, hyperprogression

## Abstract

Advances in immunotherapy have changed the therapeutic landscape of non-small cell lung cancer (NSCLC), extending overall survival over standard chemotherapy. However, by removing the protection against autoimmunity, immunotherapy can increase immune-related adverse events (irAEs). In addition, new patterns of radiological response have been observed in patients treated with immune checkpoint inhibitors (ICIs). We report the case of a 77 year-old patient with advanced lung adenocarcinoma, who presented three consecutive different irAEs (nephritis, hepatitis, and pneumonitis) and an atypical radiological response (partial response, dissociated response, and “disease flare”) in relation to treatment with the PD-1 inhibitor nivolumab. The role of ICIs in elderly patients, the incidence of consecutive irAEs, and the new patterns of radiological response, are also reviewed.

## INTRODUCTION

Cancer immunotherapy has become a new treatment approach for many oncologic diseases, including non-small cell lung cancer (NSCLC). The immune checkpoint inhibitors (ICIs) targeting the programmed cell death 1 (PD-1)/PD-ligand 1 (PD-L1) pathway, nivolumab, pembrolizumab, and atezolizumab, have shown efficacy for second-line treatment as compared with docetaxel [1]. Nivolumab has shown superior efficacy and safety to docetaxel in second-line settings in two randomized phase III clinical trials in both non-squamous (CheckMate 057) and squamous (CheckMate 017) NSCLC patients [2, 3]. Moreover, pembrolizumab is reported to have superior efficacy data compared with standard chemotherapy in first-line treatment of PD-L1 ≥ 50% tumors, with a median progression-free survival (PFS) of 10.4 months versus 6.0 months in the Keynote-024 trial [4]. Despite these encouraging results, a unique set of toxicities of ICIs has emerged, defined as immune-related adverse events (irAEs) [5, 6]. Additionally, new patterns of radiological response have also been observed in patients treated with these antibodies [7]. Here, we report the case of a patient with advanced lung adenocarcinoma, who presented three consecutive different irAEs and different radiological response in relation to treatment with the PD-1 inhibitor nivolumab. This case report addresses some of the pivotal clinical questions arising with the use of ICIs, namely the role of ICIs in elderly patients, the frequency of apparition of several irAEs for the same patient, and the new patterns of radiological response to ICIs.

## CASE PRESENTATION

**Figure 1:**
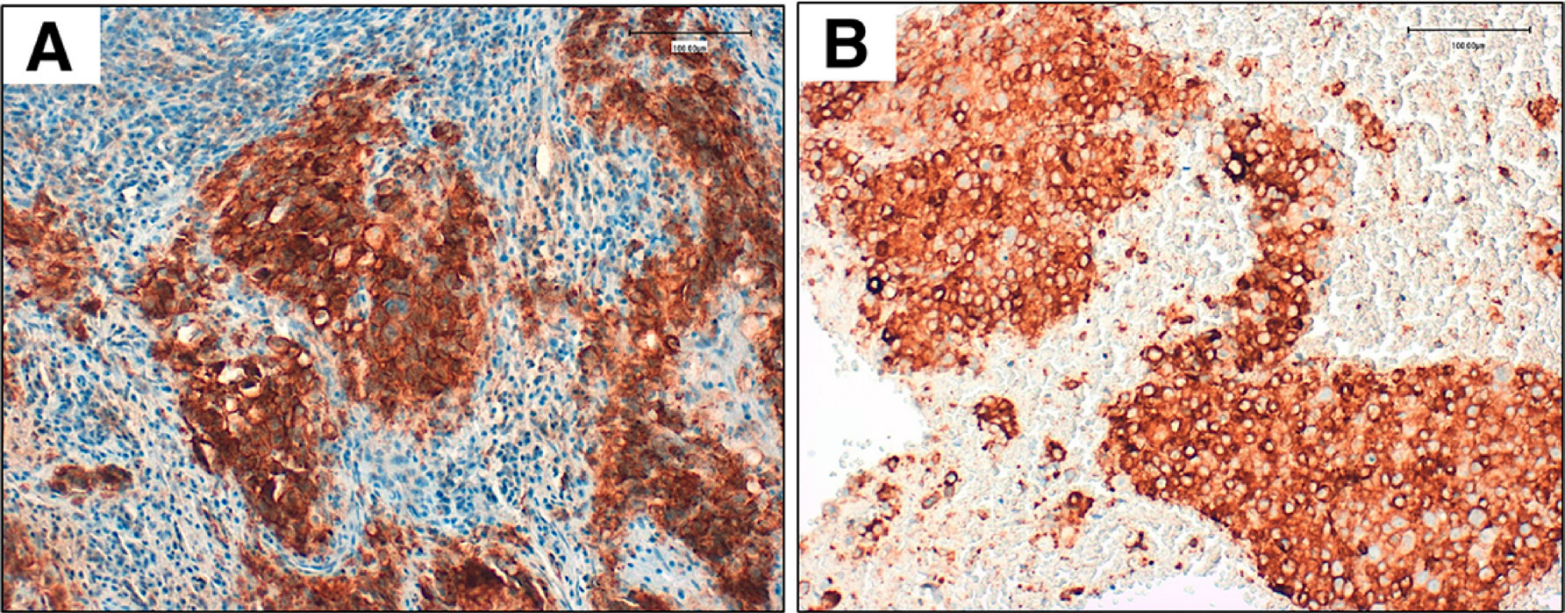
Validated PD-L1 immunohistochemical assay using clone SP263 (Ventana) on an automated staining platform (Benchmark ULTRA; Ventana) Panel (**A**) shows PD-L1 expression in the archival tumor sample (85% of expression). Panel (**B**) shows PD-L1 expression in cellular block obtained from ultrasound-guided fine-needle aspiration of the right axillary node.

A 77-year-old woman, current smoker, was diagnosed with stage IV lung adenocarcinoma and no known genetic driver mutations (EGFR, ALK, ROS1). She was treated initially with a biweekly regimen of carboplatin plus gemcitabine [8]; however, despite an initial partial response, the disease progressed after ten cycles of chemotherapy. A second-line treatment with erlotinib was administered with disease progression as best response after two months of treatment [9]. A high expression (85%) of PD-L1 by immunohistochemical staining(SP263) was detected in archival tumor samples (Figure 1A).

The patient was in good general physical condition with Eastern Cooperative Oncology Group performance status (ECOG PS) 0. The use of nivolumab became available at our center, and a compassionate use of third-line nivolumab was approved by our institution.

**Figure 2:**
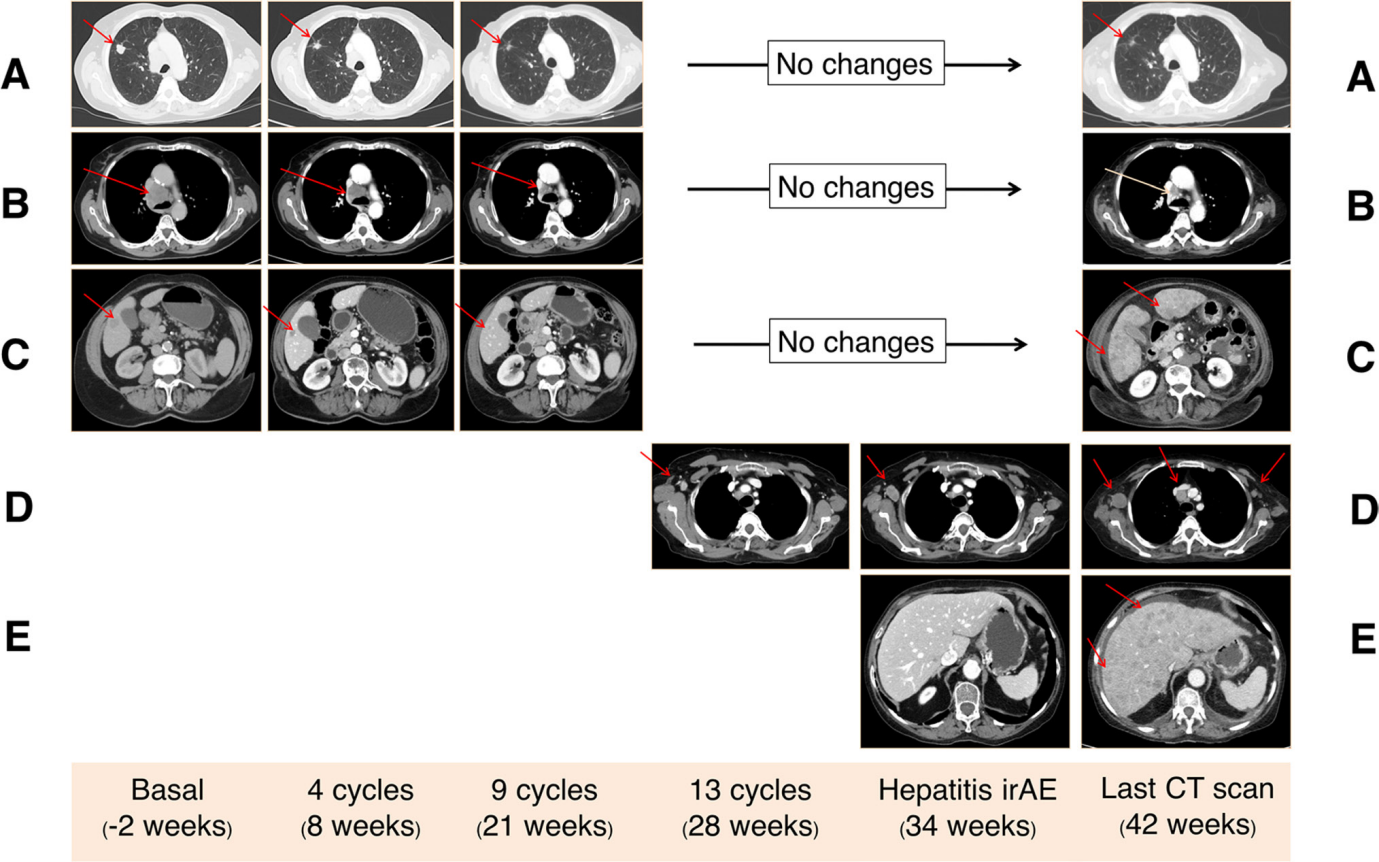
Computed tomography (CT) findings Three target lesions (red arrows) were identified in basal CT scan corresponding to lung metastasis (**A**), mediastinal lymph adenopathy (**B**), and liver metastasis (**C**). All these lesions showed a partial response after 4 cycles of nivolumab, which was maintained until the last CT scan (42 weeks). Progression of the axillary right lymph node that appeared as a new lesion at week 28 (**D**). Flare disease progression in the liver with the apparition of new uncountable liver metastasis in the last CT scan causing hepatic failure (**E**).

After 4 cycles (8 weeks from start of treatment), computer tomography (CT) scan evaluation showed a partial response in lung tumor mass, lymphatic nodes and hepatic metastasis (Figure 2A–2C). Nivolumab was well tolerated and no toxicity was observed during the seven initial cycles.

**Figure 3:**
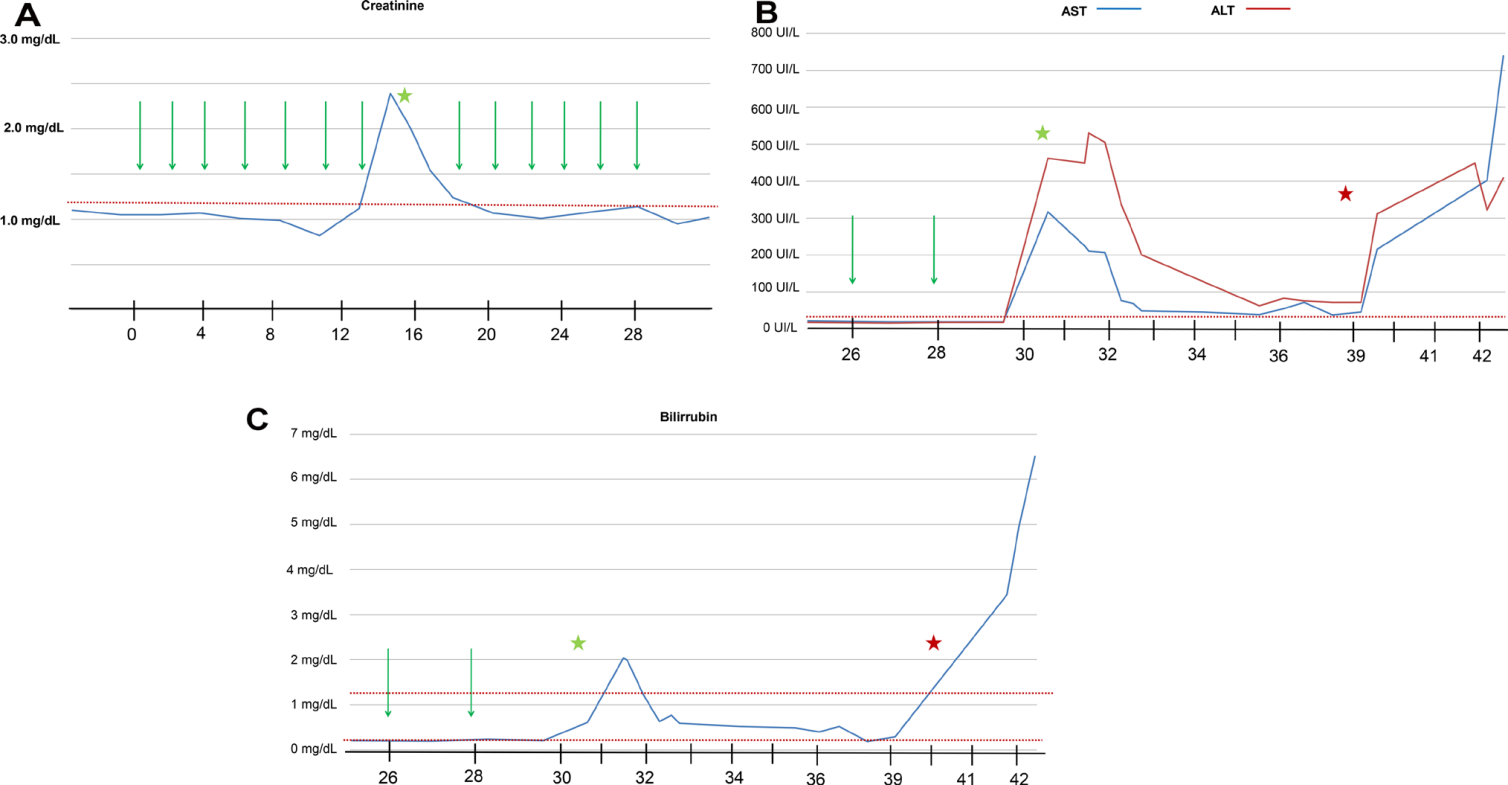
The evolution of creatinine (**A**), aspartate aminotransferase (AST) and alanine aminotransferase (ALT) (**B**), and bilirubin (**C**) levels during nivolumab treatment. Green arrow represents nivolumab treatment. Green stars represent irAE events. Red stars represent liver flare progression. Time is expressed on weeks from start of nivolumab treatment.

Before the eighth cycle (14 weeks) was started, a blood test showed an elevation in creatinine (2.39 mg/dL, previously ranged from 0.71 to1.22 mg/dL). Because an irAE (nephritis) was suspected, nivolumab treatment was stopped and methylprednisolone treatment was started (1 mg/kg/day). A 24-hour urine test dismissed nephrotoxic syndrome. Two weeks later (16 weeks), creatinine levels were lower (1.24 mg/dL) and nivolumab treatment was restarted with reduced corticoids (0.5 mg/kg/day) (Figure 3A).

Before the fourteenth nivolumab cycle (week 30), a blood test showed increased levels of liver function metrics: aspartate aminotransferase (AST), 317 UI/L (normal value <32); alanine aminotransferase (ALT), 462 UI/L (normal value <33); and bilirubin, 0.62 mg/dL (normal value <1.2), with normal values of creatinine (0.95 mg/dL) (Figure 3B, 3C). The patient was still receiving methylprednisolone at low doses (0.25 mg/kg/day) at this time. A CT scan was negative for dilatation of the biliary conduct, but a right axillary lymph node was observed, with maintained partial response of the other target lesions in the lung and liver (dissociated response) (Figure 2A–2D). Previous hepatic function had been normal, even with known hepatic metastases, which were in near radiological complete response (Figure 2C). Thus, a new irAE (hepatitis) was suspected. Nivolumab treatment was stopped, methylprednisolone dosage was increased (1 mg/kg/day) and the patient was admitted for daily monitoring of liver function. During the hospitalization period, the elevation in transaminases and bilirubin (common terminology criteria for adverse events, grade 3 and 2, respectively) had good evolution, permitting discharge at week 33. During hospitalization, methylprednisolone treatment was increased up to 3.5 mg/kg/day and the patient was discharged at a dose of 2 mg/kg/day. The patient remained completely asymptomatic (ECOG PS 0) during this time.

During an ambulatory visit at week 36, the patient reported progressive dyspnea. No relevant alterations were observed in chest radiography, and empirical antibiotic therapy (amoxicillin and clavulanic acid) was started. The patient was receiving 1 mg/kg/day methylprednisolone at this time. No clinical improvement was achieved after 5 days of antibiotics, so a new thoracic CT scan was performed, which revealed the presence of bilateral diffuse ground glass opacity suggesting nonspecific pneumonitis (data not shown). Autoimmune pneumonitis was suspected. The patient was admitted for treatment with oxygen, methylprednisolone was increased to 2 mg/kg/day, and trimetoprim/sulphametoxazol treatment was administered. To complete the study, a bronchoscopy was performed and cultures of bronchial washings were positive for Pseudomonas and Candida. An ultrasound-guided fine-needle aspiration of the right axillary node was performed during admission, which revealed malignant cells with PD-L1 expression (SP263) of 80% in immunohistochemical staining (Figure 1B). The symptoms resolved after 2 weeks treatment and the patient was discharged on 0.75 mg/g/day methylprednisolone.

**Figure 4:**
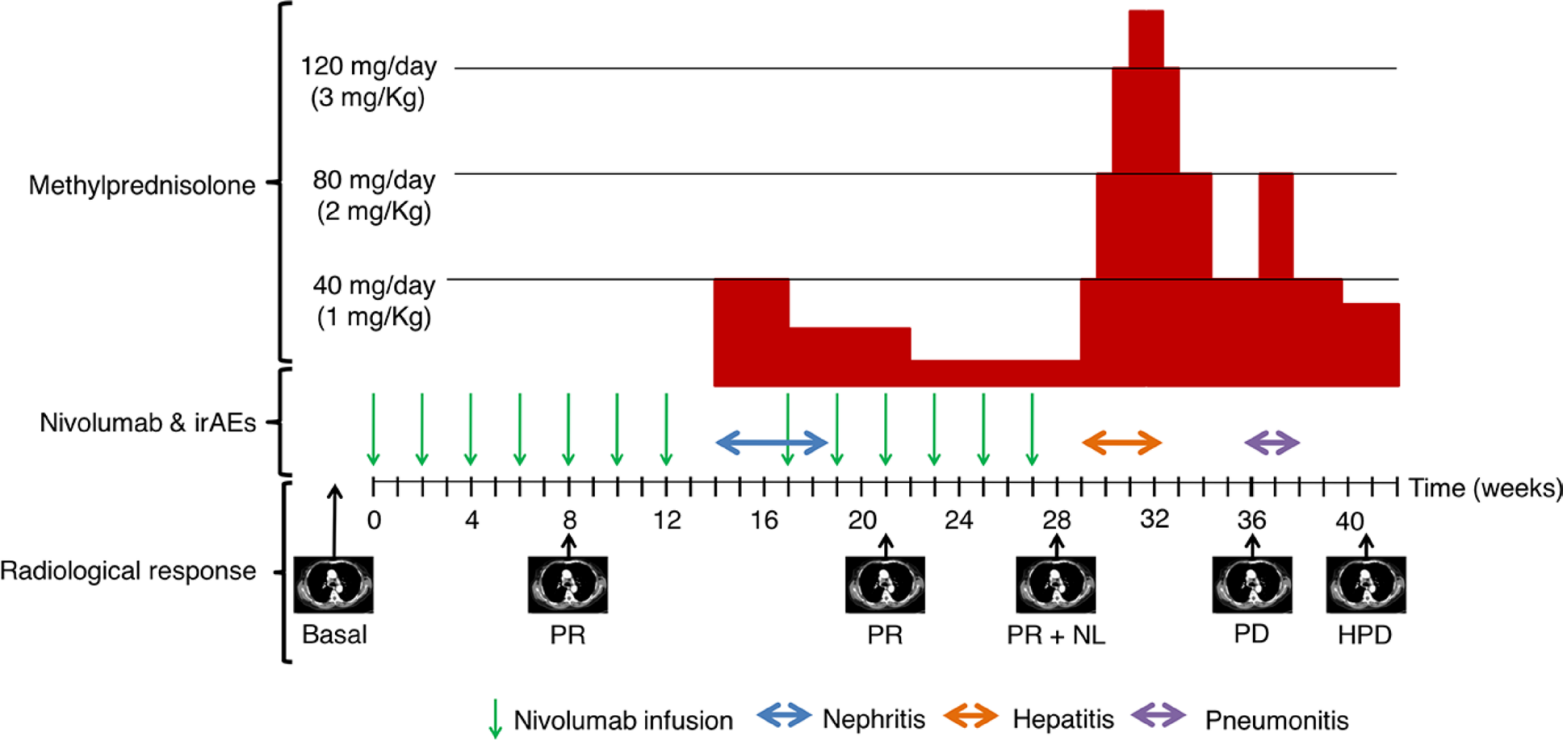
General schema of the evolution of the patient Nivolumab infusions (green arrows), the duration of the irAEs and cortisone treatment is reported. The CT scan evaluations are indicated with the radiological response: partial response (PR), new lesion (NL), progression disease (PD), and hepatic flare progression disease (HPD).

At week 40, a blood test reported elevated bilirubin and transaminases, and the patient was readmitted for study. The patient developed clinical worsening (ECOG PS 2), with flapping tremor, ascites and disorientation. Ultrasound study of the liver suggested miliary liver metastases, which were confirmed by a CT scan where multiple thoracic lymph nodes showed progression in addition to hepatic flare disease progression (initial lung metastases remained in partial response) (Figure 2E). As the patient was in a poor clinical state and in confirmed progression, best supportive treatment was initiated according to the patient and family. A graphical summary of the patient´s evolution is provided in Figure 4. A written informed consent was obtained from the patient for publication of this case report and any accompanying images.

## DISCUSSION

Immune responses are tightly regulated by signaling through co-stimulatory and co-inhibitory molecules. Cancer cells can acquire the ability to deactivate (shut-off) T-cells and suppress the immune response by presenting an immune checkpoint. The ICI drug family comprises antibodies that can physically block the checkpoint (T-cell receptor or cancer cell antigen), which frees the immune system to attack the cancer [1]. Nivolumab is a fully human IgG4 monoclonal antibody against the PD-1 receptor. By binding PD-1 receptor, nivolumab blocks the T-cell inhibition promoted by the cancer cells and their microenvironment, and thus restores the immune response.

There is a lack of high quality evidence regarding the management of cancer in the elderly, mainly due to the underrepresentation of this population in prospective randomized clinical trials. Compared with younger patients, elderly patients usually present more comorbidity, cognitive and psychological function deterioration, polypharmacy, and an increase in the need for social support. Aging is also associated with several structural and functional changes in the immune system, collectively classified under the term immunosenescence [10]. Immunosenescence is characterized by the progressive and global remodeling of immune functions during aging and is not simply a progressive decline of immune functions [11]. The advent of ICIs as a treatment for cancer patients has increased the interest in this phenomenon, and it has been suggested that immunosenescence could be associated to an increased risk of irAEs through a paradoxically higher concentration of inflammatory cytokines and autoantibodies [12].

Clinical efficacy of anti-PD1/PD-L1 in the elderly NSCLC population remains controversial. Based on small subgroup analyses from randomized clinical trials, a significant benefit of immunotherapy is observed in older patients (>65 years), with the exception of patients >75 years, who appear to obtain less benefit from ICIs [11]. Despite this, large real-life studies (from expanded access programs and retrospective cohort studies) showed a consistent comparable efficacy and toxicity profile of ICIs between young adults and patients older than 70 years. The largest ongoing study on elderly NSCLC patients is CheckMate 153, a community-based, phase 3B/4 safety study of nivolumab in patients with previously treated metastatic NSCLC in the US/Canada. In this study, no differences in responses and toxicity were observed between the two age groups of patients (<70 or ≥70 years) (Table 1) [13].

**Table 1:** Activity and adverse effects observed in the Checkmate 153 study

	<70 years	≥70 years
**N (%)**	788 (60 %)	520 (40%)
**6 months OS**	63%	63%
**AEs any grade**	59%	62%
**AEs grade 3/4**	11%	12%

Abbreviations: AEs: Adverse events; OS: Overall Survival.

In our case, the patient experienced three consecutive different suspected irAEs. A recent report has shown that in lung cancer patients treated with ICIs who experience a first irAE, and were retreated with the same ICI after recovery of the adverse event (*n* = 38), 24% of patients had the same irAE, 26% had a new irAE and 50% had no subsequent irAEs [14]. The authors also noted that irAEs were more common in patients who had experienced early-onset irAEs during initial treatment (<3 months of treatment). Therefore, this evolution can be observed in a quarter of patients that experience a first irAE and are retreated after recovery.

Radiological evolution of our patient is also of interest. Conventional response criteria, such as Response Evaluation Criteria in Solid Tumors (RECIST), were developed based on data from cytotoxic chemotherapy trials and may not be appropriate to estimate the therapeutic benefit of immunotherapy. Immune-related response criteria were therefore developed to evaluate the antitumor effects of immunotherapies: by such criteria, the appearance of new lesions or initial increase in tumor burden is not assessed as progressive disease and must be included in the total tumor burden, and progression must be confirmed via a subsequent scan [15]. Our patient initially experienced a partial response. After treatment was stopped for the second irAE (hepatotoxicity), the appearance of a new axillary adenopathy was not considered a clear progression. Immune-related unconfirmed progressive disease has been defined in the new immune-related response criteria [16], which allows atypical responses, such as delayed responses that occur after pseudoprogression, to be identified. Interestingly, the new axillary adenopathy was biopsied and we could observe that was a true pathological progression and that PD-L1 expression remained still highly positive (80%). Therefore, our patient presented a mixed response (progression on one site of the disease while other initial lesions are under partial response). Mixed response have been also described in 21.5% of patients with NSCLC treated with systemic therapy including chemotherapy or targeted therapies [17]. The incidence of mixed responses has not been well established in patients treated with ICIs, but it could be important for clinicians to decide when patients are not longer benefiting from treatment.

In addition, a new pattern of progression in cancer patients treated with ICIs has been recently described, so-called hyperprogressive disease (HPD), which is defined as disease progression by RECIST criteria with a ≥ two-fold increase in the tumor growth rate between the reference period and ICI treatment periods [18]. HPD was observed in 9% of patients, and was associated with older age (>65 years) and with worse OS. HPD has been observed at the beginning of treatment with ICIs. Recently, genetic alterations related to MDM2 family amplifications or EGFR alterations have been linked to HPD [19].

Our patient experienced a massive and very quick progression in the liver. The so-called “disease flare” has been previously described after tyrosine kinase inhibitor (TKI) discontinuation in 23% of patients with EGFR-mutant lung cancer [20]. Tumor flare reaction has also been described in some case reports of patients with ALK rearranged tumors that discontinued ALK TKI [21]. We cannot distinguish whether the flare phenomenon was merely a normal tumor progression or exhibited faster progression due to lack of treatment. Some explanation of the evolution observed in our case could relate to the prolonged interruption of nivolumab beyond the requirement of prolonged high dose of corticoids to manage immune related toxicity. One important clinical question is whether the immunosuppression mediated by corticoids, administered to reverse the irAEs, can counterbalance the expected effect of immunotherapy and explain flare progression [6]. No data from prospective studies are available that address this topic. In a retrospective analysis of patients with advanced melanoma treated with nivolumab the use of suppressive immune-modulating agents (including corticoids) for managing irAEs did not worse overall outcomes of nivolumab treatment [22]. The possibility of tumor flare reaction after discontinuation of immunotherapy could be clinically meaningful. One should acknowledge that ICIs are being used in front-line or second-line in advanced NSCLC and that currently we have response data to chemotherapy after progression to ICIs [23]. Targeting cancer with chemotherapy after failure of immunotherapy could be a valid option that may prolong survival in advanced cancer patients. In this scenario, patients who progress to immunotherapy should be identified early to allow them to be treated with other systemic treatments before cancer progression will cause a deterioration of performance status.

## CONCLUSIONS

Immunotherapy is a promising therapy that is rapidly changing the paradigms of cancer treatment. With the notable increase in their use, their associated side-effects are becoming more frequent in routine clinical practice. Understanding the role of ICIs in the elderly population is a new challenge in the context of the immunosenescence phenomenon. Moreover, new and atypical radiological response patterns need to be incorporated into our daily clinical practice.
