# Addendum: Immune-related adverse events and atypical radiological response with checkpoint inhibitor immunotherapy in an elderly patient with high PD-L1 expressing lung adenocarcinoma

**DOI:** 10.18632/oncotarget.28471

**Published:** 2023-07-01

**Authors:** Eduard Teixidor, Elia Sais, Carmen Amalia Vásquez, Walter Carbajal, Alejandro Hernández, Gloria Sánchez, Angel Izquierdo, Sara Verdura, Javier A. Menéndez, Joaquim Bosch-Barrera

**Affiliations:** ^1^Department of Medical Oncology, Catalan Institute of Oncology, Doctor Josep Trueta University Hospital, Girona, Spain; ^2^Department of Anatomical Pathology, Dr. Josep Trueta Hospital of Girona, Girona, Catalonia, Spain; ^3^Department of Radiology, Diagnostic Imaging Institute, Doctor Josep Trueta University Hospital, Girona, Spain; ^4^ProCURE (Program Against Cancer Therapeutic Resistance), Metabolism and Cancer Group, Catalan Institute of Oncology, Girona, Catalonia, Spain; ^5^Girona Biomedical Research Institute (IDIBGi), Girona, Catalonia, Spain; ^6^Department of Medical Sciences, Medical School, University of Girona, Girona, Spain

**This article has an addendum:** Patient consent was required and obtained.

Original article: Oncotarget. 2018; 9:33043–33049. 33043-33049. https://doi.org/10.18632/oncotarget.25984

